# CKD-Associated Pruritus: Updates on Management and Future Directions

**DOI:** 10.34067/KID.0000001172

**Published:** 2026-02-13

**Authors:** Kendra E. Wulczyn, Tariq Shafi

**Affiliations:** 1 Nephrology Division, Massachusetts General Hospital, Boston, Massachusetts; 2 Nephrology Division, Baylor Scott and White Health, Temple, Texas

**Keywords:** CKD

## Introduction

CKD-associated pruritus (CKD-aP), also known as uremic pruritus, is a highly prevalent and distressing symptom among people with kidney disease. CKD-aP affects approximately 51% (41%–60%) of individuals receiving dialysis and 46% (38%–55%) of persons with nondialysis CKD, with higher prevalence among those with more advanced disease.^1^ In observational studies, CKD-aP is linked to poor sleep quality, depressive symptoms, and lower health-related quality of life (HRQOL).^2^ Despite these findings, pruritus is often underreported by patients and underrecognized by nephrology providers.^3^ Encouragingly, there is now renewed attention on CKD-aP, both in clinical research and in therapeutic development. In this Perspective, we summarize contemporary approaches to clinical management, recent therapeutic advances, and priorities for future investigation.

## Clinical Assessment

The primary assessment of CKD-aP includes characterizing itch symptoms, performing a physical examination, evaluating the skin, and assessing for atypical features that might prompt consideration of a different diagnosis. Risk factors for CKD-aP, such as co-occurring systemic diseases, smoking status, and laboratory parameters, should also be considered. The Worst Itch Intensity Numeric Rating Scale (WI-NRS) is a single-question, validated instrument that provides a simple yet reliable way to quantify itch severity and monitor response to therapy.^4^ Patients are asked to rate their worst itch intensity over the past 24 hours on a zero to ten scale.

## A Simplified Approach to Management

**Figure 1:**
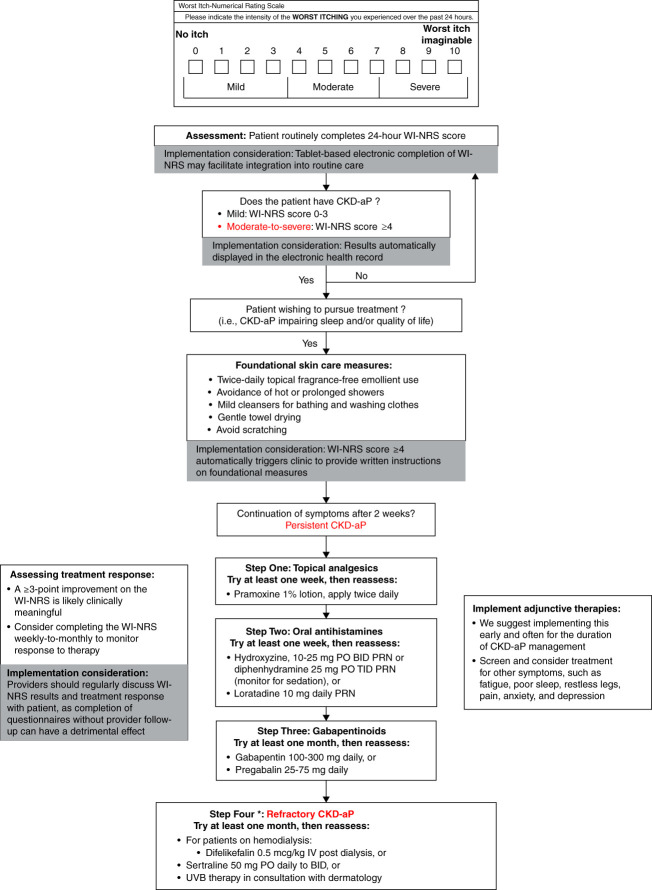
**Proposed algorithm for the assessment and management of CKD-aP.** We highlight considerations of the assessment and management algorithm in the gray boxes while acknowledging that implementation of such a protocol has not yet been studied. Persistent CKD-ap refers to symptoms lasting more than 2 weeks despite foundational skin care measures. Refractory CKD-aP denotes symptoms unresponsive to skin care measures, topical analgesics, oral antihistamines, and gabapentinoids. *Choice of treatment in step 4 depends on the availability of treatment options and patient preference. BID, twice a day; CKD-ap, CKD–associated pruritus; IV, intravenous; PO, orally; PRN, as needed; TID, three times a day; UVB, ultraviolet B; WI-NRS, Worst Itch Intensity Numeric Rating Scale.

As shown in Figure 1, management of CKD-aP begins with foundational skin care measures that address contributing factors, such as xerosis, inadequate skin hydration, and environmental irritants. Simple interventions, such as daily emollient use, avoidance of hot or prolonged showers, mild cleansers, and gentle towel drying, can meaningfully reduce symptoms.

The roles that dialysis kinetics and mineral metabolism play in the pathophysiology of CKD-aP remain uncertain, with inconsistent evidence from multiple observational studies. We suggest that optimizing Kt/V, serum phosphate, and parathyroid hormone levels be a component of all CKD-aP management; however, focusing solely on these laboratory parameters is unlikely to be sufficient to address CKD-aP in most patients.

When symptoms persist beyond 2 weeks despite optimized skin care, clinicians can pursue a stepped-care algorithm of targeted therapies shaped by insights regarding the pathophysiology of CKD-aP, which itself falls outside the scope of the current discussion but has recently been summarized by others.^5^

- Step 1: Topical analgesics. Pramoxine 1% lotion is available over the counter and can be applied to affected areas, allowing for 1 week of use to see an effect. In a small randomized controlled trial of 28 patients on hemodialysis, pramoxine applied twice daily reduced pruritus more than an emollient.^6^
- Step 2: Oral antihistamines. There are no high-quality data to support the use of oral antihistamines for CKD-aP, and providers should carefully consider the associated risk of falls and potential anti-cholinergic side effects before prescribing. However, given their low cost and ease of administration, a 1-week trial of antihistamines in thoughtfully selected patients is reasonable. Patients may prefer a nonsedating agent, such as loratadine, during the day, and a sedating agent, such as diphenhydramine, at night.
- Step 3: Gabapentinoids. Gabapentin (100–300 mg posthemodialysis or daily) or pregabalin (25–75 mg daily) are the mainstay of systemic therapy. Patients should be observed for 4 to 6 weeks to determine adequate response and should be monitored closely for the potential side effects of dizziness and somnolence. A meta-analysis of randomized controlled trials demonstrated meaningful reductions in itch from treatment with gabapentinoids.^7^
- Step 4: Options for refractory pruritus. Difelikefalin is a selective peripheral kappa-opioid receptor agonist, and its intravenous formulation was approved by the US Food and Drug Administration in 2021 for the treatment of moderate-to-severe CKD-aP in adults on hemodialysis. It is administered intravenously at the end of each hemodialysis session (0.5 *µ*g/kg), with steady-state levels achieved after the second dose. In the A Study to Evaluate the Safety and Efficacy of CR845 in Hemodialysis Patients With Moderate-to-Severe Pruritus (KALM-1) and CR845-CLIN3103: A Global Study to Evaluate the Safety and Efficacy of CR845 in Hemodialysis Patients With Moderate-to-Severe Pruritus (KALM-2) phase 3 trials (*n*=851), 51% of participants receiving difelikefalin achieved a ≥3-point reduction in WI-NRS, compared with 35% in the placebo group, with a statistically significant risk difference of 16%.^8,9^ Notably, complete resolution of itching occurred in 12% of treated patients. Significantly greater proportions of participants treated with difelikefalin also experienced meaningful improvements in itch-related HRQOL and improved sleep quality. Adverse effects from difelikefalin were typically mild, including dizziness, somnolence, and hyperkalemia. In a phase 2 trial, oral difelikefalin (1 mg daily), was effective in treating CKD-aP in patients treated with hemodialysis and those with non–dialysis-dependent CKD. However, a subsequent trial of oral difelikefalin in non–dialysis-dependent advanced CKD (NCT05342623) was terminated because of a corporate decision, and the oral formulation is not available for clinical use.

As an alternative to difelikefalin, treatment with sertraline 50 mg daily or twice daily improved WI-NRS scores after 4 weeks in two small Randomized Controlled Trials of patients on hemodialysis. Finally, for patients with persistent symptoms of CKD-aP despite the treatment options outlined above, dermatology consultation, including phototherapy with ultraviolet B irradiation, can be considered; however, it is associated with an increased risk of carcinogenesis.

Adjunctive care for pruritus should recognize the interrelatedness of symptoms experienced by patients with CKD and the effect that symptoms have on patient HRQOL. For example, CKD-aP tends to occur in a cluster of symptoms alongside poor sleep, anxiety, depression, and pain.^10^ Treating one symptom within a cluster may lead to improvement in others, and we suggest frequent screening for other CKD-related symptoms in patients with CKD-aP over the course of management.

## Emerging and Future Directions

New, targeted pharmacologic treatments for pruritus in other chronic conditions are on the horizon. As an example, inhibitors of proinflammatory interleukins, such as dupilumab, and janus kinase inhibitors show promising results in the treatment of prurigo nodularis, an intensely pruritic chronic disease. Given the likely involvement of similar signaling pathways in CKD-aP, these therapies might also prove effective for patients with pruritus and kidney disease, but clinical trials are needed. Relatedly, oral and/or subcutaneous formulations of medications are needed for the peritoneal and nondialysis CKD populations, where CKD-aP burden remains substantial.

Beyond pharmacotherapy, effective management of CKD-aP requires systematic integration into dialysis and CKD workflows. Practical steps include patient education around pruritus as a treatable symptom, assessment of itch severity at regular intervals, standardized documentation and prompts in the electronic health record, nurse-driven skin care protocols, and interdisciplinary collaboration with dermatology, social work, and mental health professionals. Implementation studies examining utilization of existing therapies, adherence, and barriers to treatment are necessary to ensure equitable care.

Areas for future study include comparative effectiveness studies comparing difelikefalin to enhanced usual care (standardized xerosis protocols plus gabapentinoids). Combination therapies also warrant investigation; given the heterogeneous mechanisms of itch, multimodal regimens, *e.g*., pairing topical, neuromodulatory, and kappa-agonist approaches, may yield additive benefit for patients with severe and refractory symptoms. Finally, given their overlapping nature, all future studies of CKD-aP treatment should also incorporate assessments of other CKD-related symptoms and HRQOL domains, rather than itch intensity alone.

## Conclusions

CKD-aP persists as one of the most underrecognized symptoms of kidney disease. Recent advances—particularly the development of difelikefalin—mark a turning point in the therapeutic landscape, allowing for targeted treatment. By embedding pruritus management within comprehensive symptom care, nephrology providers can meaningfully improve the daily lives of people living with kidney disease.
